# Whole exome sequencing identifies new susceptibility candidates underlying community-acquired pneumonia

**DOI:** 10.1016/j.gendis.2023.101170

**Published:** 2023-11-19

**Authors:** Jacobo Pardo-Seco, Sandra Viz-Lasheras, Xabier Bello, Alberto Gómez-Carballa, Alba Camino-Mera, Sara Pischedda, María José Currás-Tuala, Irene Rivero-Calle, Ana Dacosta-Urbieta, Fernando Caamaño-Viña, Carmen Rodríguez-Tenreiro Sánchez, Isabel Cifuentes, Cristina Méndez, Chiea Chuen Khor, Federico Martinón-Torres, Antonio Salas, the CAPPRIC study group

**Affiliations:** a Unidade de Xenética, Instituto de Ciencias Forenses, Facultade de Medicina, Universidade de Santiago de Compostela, and GenPoB Research Group, Instituto de Investigación Sanitaria (IDIS), Hospital Clínico Universitario de Santiago de Compostela (SERGAS), Galicia 15706, Spain; b Genetics, Vaccines and Infections Research Group (GENVIP), Instituto de Investigación Sanitaria de Santiago, Universidade de Santiago de Compostela, Santiago de Compostela, Galicia 15706, Spain; c Centro de Investigación Biomédica en Red de Enfermedades Respiratorias (CIBERES), Madrid 28029, Spain; d Translational Pediatrics and Infectious Diseases, Department of Pediatrics, Hospital Clínico Universitario de Santiago de Compostela (SERGAS), Galicia 15706, Spain; e Medical Department, Pfizer S.L.U., Alcobendas, Madrid 28108, Spain; f Genome Institute of Singapore, Agency for Science, Technology and Research, Singapore 138632, Singapore

Pneumonia is an inflammatory condition of the lung with symptoms that include productive dry cough, fever, chest pain, and difficulty breathing, and it is usually caused by viruses and bacteria, but also other microorganisms (such as fungi and parasites). Community-acquired pneumonia (CAP) is a major cause of infectious diseases, hospitalization, and mortality, especially in the elderly population.^1^ We have undertaken a case–control study on CAP patients by way of sequencing the complete exome of 300 patients and 438 healthy controls (Table S1 for demographic and clinical characteristics of patients). This study is by far the largest exome sequencing project to date aimed at exploring the potential genetic causes behind CAP.

We have sequenced the whole-exome of 300 saliva samples from CAP patients older than 18 years recruited under the umbrella of the CAPPRIC study group.^2^ Sequence analysis and statistical procedures were carried out as indicated in Supplementary Text (sequencing details are described in the previous study^3^). For the best candidate genes, we explored public resources to further investigate gene expression in COVID-19 and non-COVID-19 pneumonia patients (details in Supplementary Text).

A total of 517,502 biallelic single nucleotide polymorphisms (SNPs) were obtained after filtering indels, no-biallelic positions, monomorphic SNPs, and variants with genotyping rate >99.9%. We initially conducted a population stratification analysis by merging our dataset with 1000 Genome Project (1000 GP) population data, resulting in an overlapping set of 301,412 SNPs. Analysis of the ancestral background of samples showed the CAP exomes falling within the Western European variation, using both a multidimensional scaling analysis and a genome-ancestry analysis (Fig. 1A; Supplementary Text).

**Figure 1:**
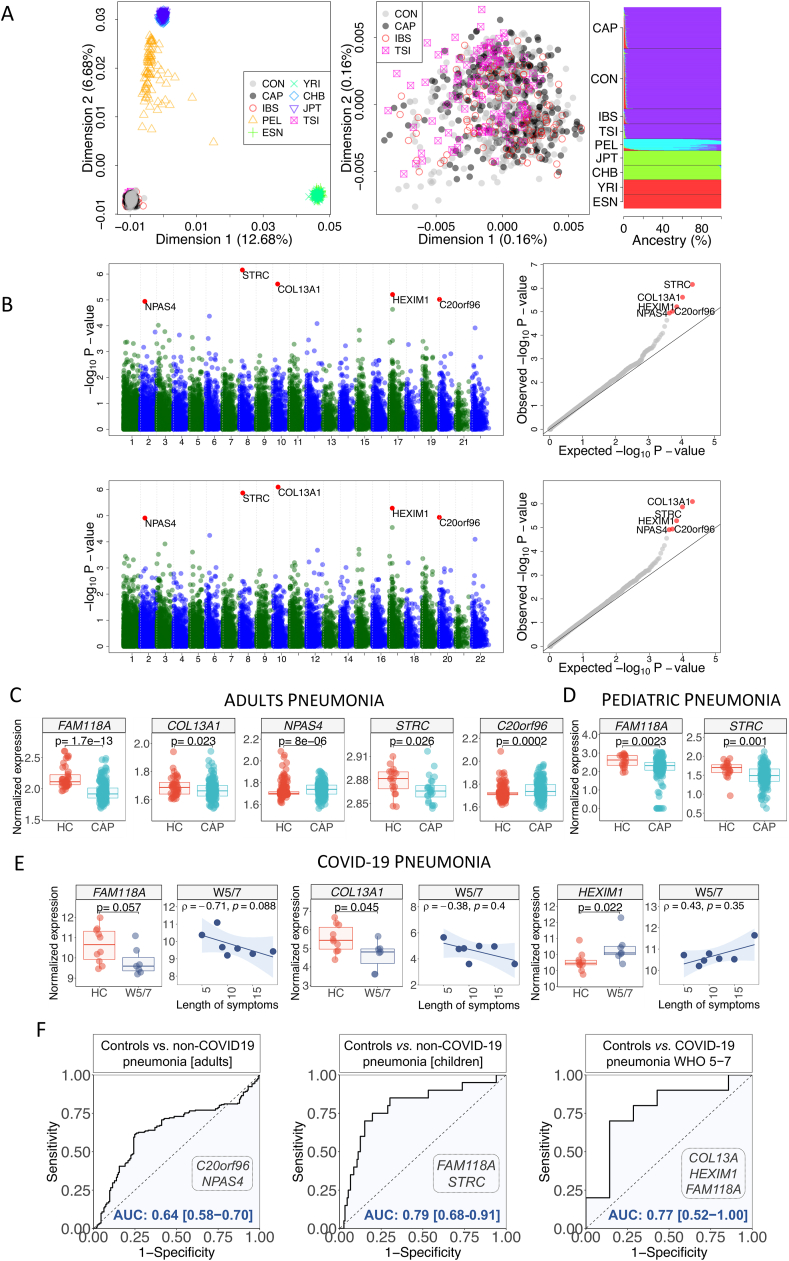
Ancestral genetic analysis of the CAPPRIC cohort, allele/gene-based association tests, and differential expression of candidate genes with best ROC-AUC predictions. **(A)** Analysis of the ancestral background of the studied cohort using reference continental population dataset from the 1000 GP. Left: Multidimensional scaling analysis for the patient's cohort and continental 1000 GP datasets; Middle: Multidimensional scaling analysis for the patient's cohort together with other European 1000 GP datasets; Right: Estimated ancestral individual values in the sampled cohort and reference 1000 GP datasets using ADMIXTURE. CAP, community-acquired pneumonia; CON, healthy controls; CHB, Han Chinese in Beijing (China); ESN, Esan (Nigeria); IBS, Iberian population (Spain); JPT, Japanese in Tokyo (Japan); PEL, Peruvians in Lima (Peru); TSI, Tuscans (Italy); YRI, Yoruba in Ibadan (Nigeria). **(B)** Gene-base association analysis using the SKAT-O collapsing association test. Manhattan plots (left) and QQ-plots (right) of *P*-values for the SKAT-O collapsing association test for all the variants in the dataset (top), and only the rare variants (minor allele frequency (MAF) < 0.05) (bottom). **(C–E)** Differential gene expression between cases and healthy controls for the best gene candidates. Only the most significant findings for each group of analysis (adult [C] and pediatric [D] non-COVID-19 pneumonia, and COVID-19 pneumonia [E]) are shown (see Table S3). In the COVID-19 dataset, we only show the severity category (WHO score > 5; W5/7). Correlation between gene expression of candidate genes and the time from symptom onset to sample collection is also displayed for the COVID-19 dataset. **(F)** ROC-AUC for the best candidate genes and the disease scenarios analyzed in the present study. Left: Non-COVID-19 pneumonia in adults; Middle: Non-COVID-19 pneumonia in children; Right: Severe COVID-19 pneumonia. We display the curves using the combination of the genes that show the best AUC-ROC values.

To carry out the single-point association test, 447,239 and 265 SNPs were removed by minor allele frequency (MAF) and Hardy-Weinberg criteria, respectively. Totally 69,998 markers survived these initial filters. Association test results revealed the most statistically significant SNPs when comparing CAP patients with controls: rs2239813, rs2753492, rs10039601, and rs700082 (Fig. S1). For the genotyping association test (additive, dominant, and recessive models), we evaluated 47,635 SNPs; being the most statistically significant one rs1056322 (Fig. S2) when considering a recessive model, which falls within the *FAM118A* gene. By further exploring the genome region closely related to this SNP, we also found two additional SNPs showing highly significant values under the same recessive model (rs6007010 and rs6007594); these three SNPs are in a region showing high linkage disequilibrium (rs1056322 *vs*. rs6007010: *r*^2^ = 0.89; rs1056322 *vs*. rs6007594: *r*^2^ = 0.76) (Fig. S3, 4). We ran the SKAT-O collapsing association tests using whole gene variability and considering only the rare variants (MAF < 0.05). Five genes passed the false discovery rate test for multiple corrections: *C20orf96*, *COL13A1*, *HEXIM1*, *NPAS4*, and *STRC* (Table S2 and Fig. 1B).

We additionally examined the expression of genes related to the most significant SNPs and genes. We used whole blood gene expression datasets available in public repositories – three of these analyzing pneumonia in adults, and another one analyzing pneumonia in children. *FAM118A* gene is significantly down-regulated in adult pneumonia patients, when compared with healthy controls (Fig. 1C and Table S3), and also in the pediatric cohort when compared with controls (Fig. 1D). Next, we examined the gene expression patterns for the five most significant genes emerging from the collapsing association tests. Except for the *HEXIM1* gene, we were able to capture the other four genes differentially expressed in some of the patient cohorts when compared with their respective controls (Fig. 1C and Table S3). The most significant findings are: i) the *NPAS4* is highly significant in the GSE65682 cohort and even more significant when meta-analyzed with the other two adult cohorts; ii) *C20orf96* is significantly up-regulated when meta-analyzed in the three adult cohorts; iii) *COL13A1* appears up-regulated only in the largest cohort GSE65682; and iv) the *STRC* gene appears significantly up-regulated in the adult cohort GSE40012 and in the pediatric cohort (Fig. 1C, D and Table S3). Analysis of the AUC (Area Under the Curve) -ROC (Receiver Operating Charasteristic) curves indicates that the combination of these candidate genes that best predicts non-COVID-19 pneumonia in adults is *C20orf96* and *NPAS4*, with an AUC-ROC value of 0.64 (0.58–0.70), while in the pediatric cohort, the combination is *FAM118* and *STRC*, with an AUC-ROC value of 0.79 (0.68–0.91) (Fig. 1F).

We also analyzed whole blood transcriptome data from pneumonia in severe COVID-19 patients (with WHO score values between 5 and 7 (W5/7)). Two candidate genes were found to be expressed differentially in cases and controls (Fig. 1E and Table S3). The *FAM118A* gene was found to be down-regulated in severe COVID-19 cases when compared with healthy controls. Most interestingly, the expression of this gene decays with the time from symptom onset (Spearman's *ρ* = −0.71). The *HEXIM1* gene was up-regulated in the most severe patients when compared with healthy controls. Most remarkable is that the expression of *HEXIM1* appears to increase with the time from symptom onset (Spearman's *ρ* = 0.43; Fig. 1E). The *COL13A1* gene is however down-regulated in the most severe patients; the expression of this gene decays over time from symptom onset (Spearman's *ρ* = −0.38; Fig. 1E). Analysis of the AUC-ROC curves indicates that the combination of genes (among the best candidates) that best predicts severe COVID-19 pneumonia is *COL13A1*, *FAM118A*, and *HEXIM1*, with an AUC-ROC value of 0.77 (0.52–1.00) (Fig. 1F).

Statistical association tests on allele and genotype frequencies, and the analysis of collapsing variants per gene in cases and controls, have allowed to shed light on new candidate susceptibility variation to pneumonia. Single-allele association tests highlight the variant rs1056322, located in the 3'UTR at "the family with sequence similarity 118 member A" (*FAM118A*) gene as the most significant one (under a recessive model), with two other closely related SNPs that are in high linkage disequilibrium and show suggestive statistical significance, namely rs6007010 (synonymous SNP falling in the neighboring gene *SMC1B*) and rs6007594 (non-synonymous variant located in *FAM118A*). The association of *FAM118A* with pneumonia is unclear (Supplementary Text) and needs to be validated in an independent cohort. The most remarkable findings of the present study come from exploring the pathogenicity accumulated in single genes; the most significant ones being *COL13A1*, *C20orf96*, *HEXIM1*, *NPAS4*, and *STRC*. The main interest of these candidates is that none of them has been previously signaled as possible candidates for pneumonia; this may be explained, at least in part, by the fact that our study is based on whole exome sequencing, in contrast to previous studies that focused on candidate genes or GWAS approaches. At least, *COL13A1*, *HEXIM1*, and *NPAS4*, have been previously reported to be involved in respiratory-related disorders (Supplementary Text). By exploring gene expression of the top genes in available datasets of non-COVID-19 and COVID-19 pneumonia patients, we found supporting evidence for these new candidates. AUC-ROC curves of gene expression values were evaluated for the most significant genes. We found that for both severe COVID-19 and non-COVID-19 pneumonia conditions, combinations of these genes might jointly predict the disease outcome (Fig. 1F). This adds indirect evidence supporting the involvement of these genes in the pneumonia phenotype. The cohorts and datasets analyzed do not allow us to determine whether the statistical associations observed are specific to the European population. In the case of exome data, the primary findings were derived from a gene-based test that assesses variation by aggregating SNP variations. When it comes to gene expression data, it is not possible to infer genetic background from microarray data.^4^ Although with limitations (Supplementary Text), we have gathered reasonable genomic evidence at the gene association and gene expression levels that provides a suggestive association between these genes and their contribution to pneumonia susceptibility.

Our exploratory analysis of genome-wide susceptibility to CAP has revealed a few new gene candidates. The causal link between these candidates and the disease is unclear, but the suggestive statistical association evidence, coupled with consistent gene expression data, opens new research scenarios that need further investigation.

## Ethics declaration

The study was conducted according to the guidelines of the Declaration of Helsinki, and approved by the Institutional Regional Ethics Committee of Madrid (CEIC-R) with the reference PFI-PRE-2015-01 on 12 January 2016. Informed consent was obtained from all subjects included in the study.

## Author contributions

F.M.-T., I.C., and C.M. contributed to the conception and design of the work; The CAPPRIC study group contributed the samples and performed the acquisition of the clinical data and the follow-up; I.R.-C., A.D., F.C.-V., and C.R.-T. contributed to the interpretation of the clinical data; J.P.-S., S.V.-L., X.B., A.G.-C., A.C.-M., S.P., M.J.C.-T., and A.S. analyzed the sequence genome data; J.P.-S. and A.S. drafted the manuscript; all authors reviewed the manuscript critically for important intellectual content and provided final approval of the version to be published. All authors read and agreed to the published version of the manuscript.

## Conflict of interests

F.M.-T. reports grants to their institutions from Pfizer S.L.U., Madrid, Spain for this study, and he has received honoraria from GSK, Pfizer S.L.U., Sanofi Pasteur, MSD, Seqirus, and Janssen for taking part in advisory boards and expert meetings, and for acting as speaker in congresses outside the scope of the submitted work. F.M.-T. has also acted as principal investigator in RCTs of the above-mentioned companies, and Ablynx, Regeneron, Roche, Abbott, Novavax, and MedImmune, with honoraria paid to his institution. I.R.-C. has participated in advisory boards organized by MSD, GSK, Sanofi, and Pfizer. I.R.-C. has been involved in clinical trials funded by Ablynx, Abbot, Seqirus, Sanofi Pasteur MSD, Cubist, Wyeth, Merck, Pfizer, Roche, Regeneron, Jansen, Medimmune, Novavax, Novartis, and GSK, with the funds were awarded to the institution; A.D. received funds (awarded to the institution) by participating as researcher of GSK and Pfizer S.L.U. pneumococcal vaccines clinical trials. I.C. and C.M. are employees of Pfizer S.L.U., Madrid, Spain.

## Funding

This study was sponsored by Pfizer. It also received support from Instituto de Salud Carlos III ([ISCIII] TRINEO: No. PI22/00162; DIAVIR: No. DTS19/00049; Resvi-Omics: No. PI19/01039 (to A.S.); ReSVinext: No. PI16/01569; Enterogen: No. PI19/01090 (to F.M.-T).; cofinanciados FEDER, GAIN: Grupos con Potential de Crecimiento No. IN607B 2020/08 and Grupos de Referencia Competitiva No. IN607A 2023/02 (to A.S.); ACIS: BI-BACVIR No. PRIS-3 (to A.S.), and CovidPhy No. SA 304C (to A.S.); consorcio Centro de Investigación Biomédica en Red de Enfermedades Respiratorias No. CB21/06/00103 (to F.M.-T.); GEN-COVID No. IN845D2020/23 (to F.M.-T.) and Grupos de Referencia Competitiva No. IIN607A2021/05 (to F.M.-T).
