# Supplementary material for: Whole exome sequencing identifies new susceptibility candidates underlying community-acquired pneumonia
Source: Genes Dis. 2023 Nov 19;11(6):101170. doi: 10.1016/j.gendis.2023.101170 (PMC11327392; doi:10.1016/j.gendis.2023.101170)
Supplement: Multimedia component 1 [file mmc1.docx]

*Supplementary Text*

**1. Additional background**

According to Torres et al. ^1^, the estimated incidence in Western countries is 1.5–1.7 cases per 1,000 population, reaching 14 per 1,000 person-years in population ≥65 years old. There are a few studies aimed at determining the etiology of CAP using different laboratory methods. The study by Juvén et al. ^2^ determined that, in a cohort of 254 hospitalized CAP patients, 62% of pneumonias were due to a viral infection, whereas a bacterial agent was detected in 53% of the cases; a concomitant viral-bacterial infection was detected in 30% of the patients. *Streptococcus pneumoniae*, respiratory syncytial virus (RSV) and rhinovirus are the most common causal pathogens in CAP.

As observed in many infections ^3-5^, a number of mutations in the host have been reported to be associated to host response to infections that cause pneumonia, e.g. related to antigen recognition (CD14, toll-like receptors, etc.), proinflammatory and anti-inflammatory responses (TNF-α, interleukin-6, interleukin-10, etc.), and effector mechanism (angiotensin-converting enzyme, coagulations system genes, etc.); reviewed in Waterer and Winderink ^6^. Several of these studies focused on candidate genes, showing both negative findings with the analysis of polymorphisms at the *FCN2* gene ^7^, and positive associations at polymorphisms in the *NFKBIL2* gene ^8^. Using a genome-wide association study (GWAS), Anderson et al. ^9^ analyzed an Australian pregnancy cohort in regards to the pneumococcal surface protein C vaccine against *S. pneumoniae*; they found a few relevant polymorphisms and haplotypes at the HLA complex that might have a profound influence over the ability of infected children to develop antibody response to *S. pneumoniae* and to pneumococcal protein vaccines. More recently, we carried out a whole exome sequencing study (WES) of selected patients with confirmed diagnosis of pneumococcal pleural effusion (empyema) ^4^; the study identified new candidate genes, namely *MEIS1*, *OR9G9*, *MUC6*, *MUC3A* and *APOB*, all of them statistically associated with this complicated form of pneumococcal pneumonia. Moreover, a large-scale meta-analysis carried out on the UK Biobank and FinnGen ^10^, found two independent SNPs (rs2009746 and rs76474922) associated with pneumonia, and 18 candidate genes (including *IL127*, *PBX3*, *APOBR* and *CHRNA3/5*). The study by Chen et al. ^11^ on the Vanderbilt University biobank detected two regions of large effect, the CFTR and the HBB locus. Most recently, several additional studies have been carried out on COVID-19 pneumonia; these highlighted the role of inborn errors of type I interferons (IFNs), including autosomal TLR3 and X-chromosome-linked TLR7 deficiencies ^3;12^, and pre-existing auto-antibodies neutralizing IFNα, IFNβ and/or IFNω, as reviewed in Zhang et al. ^13^

**2. Supplementary material and methods**

Sampling

The CAPPRIC study group is a multi-center active surveillance study aiming at evaluating the main characteristics of CAP in patients older than 18 years, all with confirmed diagnosis of pneumonia by chest radiography, attending to 24 Spanish primary care centers ^14^. The CAPPRIC exome study cohort consists of 300 saliva samples collected from patients who suffered from CAP, all of them belonging to a larger cohort of patients analyzed in Molina et al. ^14^. Urine samples were collected for pneumococcal antigen testing (Binax Now®), detecting 11 positive infected patients (the other samples being negative). The patients’ main clinical characteristics are summarized in **Table S1**. As control cohort we considered 438 samples taken from healthy donors (47 venous blood sample and 391 saliva samples); there is no evidence indicating an impact on statistical analysis due to the different biological origin of the control samples (e.g. there is no outliers observed in the MDS plot of **Figure 1A**). Saliva samples were collected from patients in Oragene OG-500 devices (DNA Genotek) and DNA was isolated using the prepIT L2P kit (DNA Genotek), following the standard protocol. DNA from blood samples was isolated using Wizard Genomic DNA Purification Kit (Promega) and following the recommended protocol.

Sequencing process

We carried out whole-exome sequencing and targeted sequencing in all cases and healthy controls. Libraries were prepared using hybridization capture kits (Roche-Nimblegen SeqCap) ^15^.

Details on sequencing process and variants calling were previously described in Li et al. ^16^ and are summarize here. Raw DNA sequence data from Illumina's HiSeq4000 and NovaSeq6000 instruments were employed. First, raw DNA sequence reads were aligned to the hg19 genome reference. Duplicated reads, indicating potential errors, were flagged, and removed. Variant detection focused on exons and the 50 base-pair regions around them. To ensure data integrity, all data were uniformly processed. Unaligned sequence reads were aggregated into BAM files using PICARD, BWA, and GATK software, following best practice guidelines (https://www.biorxiv.org/content/10.1101/201178v3). Genetic variants were identified and genotyped using recalibrated BAM files with the Haplotype Caller from GATK. This process occurred in batches of 200 samples per batch. All genetic variants were annotated using the GRCh37 (equivalent to hg19) database through the Ensembl Variant Effect Predictor. Annotations included details about functional effects such as synonymous, non-synonymous, splice-acceptor, splice donor, insertions-deletions causing frame shifts, start loss, stop loss, and stop gain. For variants with multiple effects across different transcripts, the highest impact effect was selected. Quality control measures were rigorously applied. Variants needed a minimum sequencing depth of 10x coverage and a call rate of at least 95%. Samples were stratified by the country of recruitment. Variants showing significant deviations from Hardy-Weinberg Equilibrium and differences in completion rates between cases and controls were excluded. Multi-allelic variants were managed as collections of independent bi-allelic variants. Additionally, genetic variants were required to have an allelic balance between 20% and 80% to minimize false identifications. Variants passing quality checks were subject to a variant quality score log-odds (VQSLOD) cutoff, ensuring high sensitivity. VQSLOD scores were derived from variant quality score recalibration (VQSR). Further quality control checks removed outlier samples, ensuring reliable association tests. Sequencing depth requirements were stringent, with samples needing >80% of targeted bases sequenced to 20X or more. Samples with discordant ancestry and sex, duplicates, and related samples were excluded. Additional scrutiny eliminated samples with an unusually low or high number of singletons, which could indicate DNA contamination or low quality. Concordance with GWAS arrays was checked for most samples, and high concordance (>99%) was observed across all variants for both discovery and replication exome sequencing stages.

Statistical analysis

First, we carried out a curating process, removing non-autosomal variants, indels, and variants with a genotyping rate below 99%. To reduce the false positive rate, we carried out analyses aimed at detecting samples with different ancestral population background, as done previously ^4;17^. For this purpose we considered 1000 Genome Project (henceforth 1000GP) populations as reference dataset, sub-selecting populations of the four main ancestry origins: African (Yoruba in Ibadan, Nigeria, YRI; and Esan in Nigeria, ESN), European (Iberian Population in Spain, IBS; and Toscani in Italy,TSI), East Asian (Han Chinese in Bejing, China, CHB; and Japanese in Tokyo, Japan, JPT) and mixed American (Peruvians from Lima, Peru, PEL).

We then used Identity-By-State (IBS) values to build multidimensional scaling (MDS) plots enabling the detection of possible genome outliers. Maximum likelihood estimation of individual ancestries from multi-locus SNP data was carried out using the software ADMIXTURE ^18^.

Next, we carried out single-point SNP association tests under different genetic models, to explore if there are genetic factors that could explain the studied phenotype. Statistical tests were carried out first on alleles, and subsequently on genotypes considering an additive, a dominant, and a recessive model. Single-point Fisher’s exact test was carried out for allelic and genotype-based association analysis between the CAP cohort and the healthy controls. Allele statistical association tests were computed only for those variants with Minor Allele Frequency (MAF) above 0.05 and Hardy-Weinberg *P*-value >0.001 for both case and control cohorts. For all genotype-based model association analysis, only SNPs having >5% for all genotype categories were considered.

To investigate the effect of accumulated pathogenic variants in genes on the pneumonia phenotype, we performed a statistical association test using the collapsing method known as the Optimized Sequence Kernel Association Test (SKAT-O) ^19^. The SKAT-O test combines a set of generalized SKAT tests using different proportions of SKAT *vs*. Burden. For this purpose, we considered the annotated information of each variant extracted from the Combined Annotation Dependent Depletion (CADD) v1.6 database ^20^, which includes 57,118 genes. We undertook statistical analysis considering: *i*) all variants in genes, and *ii*) only the rare variants (MAF < 0.05). Only genes with at least ten variants were considered for these analyses.

Plink v1.9 ^21^ software was employed to curate the data and perform the single-point analysis. R statistical software ^22^ was used for most computations and graphic representations, particularly the *LocusZoom* R script ^23^ for LD/*P*-value representation for single-point analysis, and *gaston* R library ^24^ for LD pairwise plot. The *SKAT* R library ^25^ was used to compute the SKAT-O gene-based association tests. A nominal significance level was set to 0.05. To monitor multiple test, we used the False Discovery Rate (FDR) approach.

Analysis of gene expression data

We examined transcriptomic data available in public repositories to further explore expression patterns of the best gene candidates inferred from our WES data. We retrieved from the public repository Gene Expression Omnibus (GEO) four microarray expression datasets examining patters of gene expression in whole blood samples of pneumonia patients: a) GEO acc. No. GSE65682, including 40 healthy control and 194 adult pneumonia cases ^26^, (b) GSE42834, including 113 healthy controls and 6 adult pneumonia cases ^27^; (c) GSE40012, including 18 healthy controls and 24 adult pneumonia cases ^28^; and (d) GSE103119, including 20 healthy controls and 152 pneumonia cases from a pediatric cohort ^29^. The datasets GSE65682, GSE42834, and GSE40012 were analyzed jointly, while the GSE103119 dataset was analyzed independently because it only considered pediatric cases.

We normalized each dataset separately with the package *Oligo* ^30^ for Affymetrix® datasets and *limma* ^31^ for Illumina® microarrays datasets. We used the R package COCONUT (COmbat CO-Normalization Using conTrols) to combine the three datasets and reduce batch effects in the analysis ^32^.

In addition, from the functional genomics data collection ArrayExpress repository (https://www.ebi.ac.uk/biostudies/arrayexpress) we downloaded the MTAB-10926 dataset, which contains gene expression RNA-seq data of COVID-19 pneumonia patients stratified by severity ^33^; we only analyzed the samples collected from patients displaying a severe phenotype (measured as having a WHO score value between 5 and 7 [W5/7]) of pneumonia). We also included new RNA-seq data from 10 healthy controls (unpublished data) sequenced together with the samples from Jackson et al. ^33^ to be used as a comparison group.

The statistical differences between case-control groups for each candidate were evaluated using test Wilcoxon test; meanwhile the correlation between expression levels and days of symptoms was assessed using the Spearman’s ρ coefficient.

We computed the Receiver Operating Characteristic (ROC) curves and the Area Under the Curves (AUC) for the set of the most significant candidate genes using the *pROC* package ^34^. The AUC confidence intervals (95%) were calculated through a stratified bootstrap resampling.

Additionally, we explored genome-wide co-expression correlations to predict gene functions and explore gene-gene interactions using the *CorrelationAnalyzeR* package ^35^. We used the different immune-related datasets from normal conditions (>12K) available in the package as reference for the inferences. Using the reference database the package can find the top diverging and top similar co-expression correlations between two genes of interest not only at a gene level but also at a pathways level.

**3. Additional results**

*Analysis of ancestral background*

The two main dimensions of the MDS plot (**Fig. 1A left panel in main text**) show a triangular pattern, with the main continental ancestries displayed in the three vertexes, and native American genome profiles (PEL) occupying intermediate positions between the Asian and the European clusters. Dimension 1 separates the African datasets (ESN and YRI) from the European (IBS and TSI) and East Asian (JPT and CHB) ones, and it accounts for 12.68% of the total variability. The main effect of Dimension 2 is to separate the East Asian datasets from the Sub-Saharan and European ones, and it accounts for 6.68% of the total variability. The CAP cohort and the healthy controls genome profiles fall fully in the European pole of the MDS (**Fig. 1A left panel in main text).** As shown in a more detailed MDS analysis focused on our studied samples and two other European ancestry samples (IBS and TSI; **Fig. 1A center panel in main text**), both cases and controls have a comparable genome background that fits with that of other European sample sets.

The pattern observed in the MDS plot agrees well with admixture analysis (**Fig. 1A right panel in main text**), indicating that the CAP cohort shares most of its ancestry with the other European ancestry population sets used in the model (97.8% European ancestry, 0.5% East Asian ancestry and 1.7% Sub-Saharan ancestry) in a comparable proportion to the control cohort (99.1% European ancestry, 0.4% East Asian ancestry and 1.0% Sub-Saharan ancestry). The IBS sample set (Iberian population in Spain) is the closest 1000GP group to the pneumonia samples (98.4% European ancestry, 0.5% East Asian ancestry, 1.7% Sub-Saharan ancestry).

*SKAT-O association test*

We found 21,511 genes fulfilled the criteria of containing at least 10 SNPs; 19,987 of them had MAF < 0.05. We ran the SKAT-O collapsing association tests using whole gene variability and considering only the rare variants (MAF < 0.05). Five genes passed the FDR test for multiple correction when considering the whole gene variability (**Table S2**; **Fig. 1C upper panels in main text**): *C20orf96* (*P*-value = 9.55×10^-6^; FDR *P*-value = 4.89×10^-2^), *COL13A1* (*P*-value = 2.43×10^-6^; FDR *P*-value = 2.61×10^-2^), *HEXIM1* (*P*-value = 6.18×10^-6^; FDR *P*-value = 4.42×10^-2^), *NPAS4* (*P*-value = 1.14×10^-5^; FDR *P*-value = 4.89×10^-2^), and *STRC* (*P*-value = 6.95×10^-7^; FDR *P*-value = 1.49×10^-2^). Comparable significant values were obtained when applying SKAT-O only on the rare variants (MAF < 0.05): *C20orf96* (*P*-value = 1.15×10^-5^; FDR *P*-value = 4.90×10^-2^), *COL13A1* (*P*-value = 8.03×10^-7^; FDR *P*-value = 1.35×10^-2^), *HEXIM1* (*P*-value = 5.17×10^-6^; FDR *P*-value = 3.43×10^-2^), *NPAS4* (*P*-value = 1.23×10^-5^; FDR *P*-value = 4.90×10^-2^), and *STRC* (*P*-value = 1.35×10^-6^; FDR *P*-value = 1.35×10^-2^); (**Table S2**; **Fig. 1C, lower panels in main text)**.

*Co-expression of FAM118A and HEXIM1*

We further investigated the functional immunological implications of the differential expression between *FAM118A* and *HEXIM1* genes (**Table S3**) found in COVID-19 pneumonia using a co-expression correlation approach. The top biological processes showing the highest differences in activity (measured as Normalized enrichment score [NES]) between both genes were ‘DNA geometric changes’ (NES variance = 4.53) and ‘DNA dependent DNA replication’ (NES variance = 4.63) (**Table S4**; **Figure S5**). The *FAM118A* individual NES was significantly positive for both pathways (*NES_FAM118A_* = 1.54 and 1.69 respectively) pointing to an up-regulation of *FAM118A* in normal immune tissues for these processes, whereas *HEXIM1* showed an inverse pattern (NES_HEXIM1_ = -1.47 and -1.35, respectively) (**Table S4**; **Figure S5**).

However, most of the significant pathways detected yielded very similar and positive NES values between both genes (**Table S4**). Among the top 30 similar pathways (lowest NES variance) an over-representation of terms related to epigenetic regulation, more specifically with histone methylation, were found. Thus, from these top similar pathways those showing the highest NES average (NES > 2) were ‘heterochromatin organization’, ‘regulation of histone methylation’, ‘negative regulation of gene expression, epigenetic’ and ‘histone methylation’.

**4. Additional discussion**

*C20orf96*

Two intron variants, rs6035505 and rs59953878, located at the chromosome 20 open reading frame 96 (*C20orf96*) have been reported to be associated with COVID-19 ^36^; also recorded in the GWAS catalog. Unfortunately, this gene codes for an unknown protein for whose biological function there is no information available. This gene binds to transcription factor binding sites AREB6, GATA-1, GATA-2, GATA-3, ATF6, c-Myc, Max, CHOP-10, AMLa, and C/EBPalpha (GenCards; https://www.genecards.org). We observed that this gene is up-regulated in pneumonia patients, adding further evidence for its involvement in this disease condition (Wilcoxon test *P*-value = 0.0002) (**Figure 1C, fifth panel, in main text**).

*COL13A1*

The ‘collagen type XIII alpha 1 chain’ (*COL13A1*) gene is cataloged several times in DisGeNET ^37^ as ‘respiratory tract disease’ and ‘Infections; Respiratory Tract Diseases’, among many other non-respiratory disease conditions. This gene has been studied in the context of hyperbaric oxygen (HBO) in a model of hyperbaric oxygen-treated mice lungs. HBO is widely applied to treat several hypoxia-related diseases ^38^; this technique, however, has important side effects, with oxygen toxicity in the lung being the main one. These authors have found the up-regulation of *COL13A1* matrix fibroblast to be relevant in response to the toxicity generated by HBO and in general to the fibrotic pathogenesis. Increased levels of *COL13A1* have also been reported to be relevant in the context of pulmonary fibrosis in a human spheroid model aimed at recapitulating drug-induced pulmonary fibrosis ^39^. By further investigating *COL13A1* in several gene expression datasets, we observed that this gene is significantly down-regulated (Wilcoxon test *P*-value = 0.023) in non-COVID-19 patients (**Figure 1C, second panel, in main text**), and in severe COVID-19 pneumonia patients (Wilcoxon test *P*-value = 0.045) when contrasting cases vs. controls (**Figure 1E, third panel, in main text**); for these COVID-19 cases, the expression decays with the time from onset of symptoms (**Figure 1E, fourth panel, in main text**).

*FAM118A*

The SNP rs1056322, located in *FAM118A*, has been found to be the most statistically associated in our cohort of patients when compared to controls. It has been found to be associated with several disease conditions (as recorded in the GWAS Catalog; https://www.ebi.ac.uk/gwas/) e.g. pulse pressure ^40^, or chronic inflammatory diseases ^41^. The link between *FAM118A* and infectious diseases is unclear. Gardinassi ^42^ has recently reported this gene to be associated to human bronchial epithelial cells infected with respiratory syncytial virus. We have found that *FAM118A* gene expression is significantly down-regulated in blood samples from pneumonia pediatric patients (Wilcoxon test *P*-value = 0.0023) (**Figure 1D, first panel, in main text**), and highly significant in pneumonia adult patients (Wilcoxon test *P*-value = 1.7×10^-13^) (**Figure 1C, first panel, in main text**).

*HEXIM1*

The ‘hezamethylene bis-acetaide inducible protein 1’ (*HEXIM1*) gene induces hexamethylene-bis-acetamide in vascular smooth muscle cells. *HEXIM1* is a role player in several immunological processes. For instance, *HEXIM1* takes part in the regulation of DNA virus-mediated innate immune response, by assembling into the HDP-RNP complex ^43;44^. The HEXIM1 protein inhibits the kinase activity of P-TEFb (CDK9/cyclin T), which is indispensable for the replication of human immunodeficiency virus type 1 (HIV-1), since it is a specific host cellular cofactor for the viral Tat protein ^45;46^. It has also been reported that the cardiomyocyte-specific overexpression of *HEXIM1* prevents right ventricular hypertrophy (RVH), and it could be a good therapeutic target for RVH, according to Yoshikawa et al. ^47^. The common *HEXIM1* synonymous variant rs1044977 (effect allele C) has been found to be associated with childhood onset asthma, as recorded in the GWAS catalog, in a genome-wide genotyping study performed in UK Biobank participants ^48^ that used a discovery sample of >13K European ancestry cases and 393K controls. In our cohort, this SNP is statistically significant under the recessive and additive models (Recessive model: genotyping frequencies in cases: 0.110/0.890 vs. controls 0.057/0.943, *P*-value = 1.17×10^-2^; additive model: genotyping frequencies in cases: 0.110/0.323/0.567 vs. controls 0.06/0.41/0.53, *P*-value = 6.48×10^-3^), with the alternative allele being C. By exploring *HEXIM1* expression we have also found that this gene overexpresses more clearly in the most severe COVID-19 pneumonia patients (Wilcoxon test *P*-value = 0.022 when comparing cases *vs*. controls) (**Figure 1E, fifth panel, in main text**); also remarkable is the observation that the gene expression of *HEXIM1* in these patients increases with the time from onset symptoms (although this trend is not statistically significant, maybe due to the limited number of samples considered) (**Figure 1E, sixth panel, in main text**). Considering the role of this gene in the innate immune response, this observation recalls the known impairment of the innate immune response observed in severe COVID-19 patients ^33;49^.

*NPAS4* gene

The ‘neural transcription factor genes’ *NPAS4* and, in general, the NPAS gene family, has been reported to be strongly down-regulated in the context of the Zika-induced neuronal dysfunction ^50^, with evidence at both the gene expression and the protein level. The SNP rs76422502 (a non-coding transcript exon variant) is recorded in the GWAS catalog as being highly statistically associated with lung function in vital capacity (meaning the volume of air that is exhaled by a maximal expiration following a maximal inspiration); this evidence comes from a study that leverages polygenic functional enrichment to incorporate coding, conserved, regulatory, and LD-related genomic annotations into association analyses ^50^. Further studies would be needed to better understand the possible link of *NPAS4* gene in pneumonia with the role that this gene plays as a main contributor to neuronal dysregulation produced by Zika virus infection. Regarding to gene expression, we observed that NPAS4 is significantly overexpressed in CAP adults patients in comparison with healthy controls (Wilcoxon test *P*-value = 8×10^-6^) (**Figure 1C, third panel, in main text**).

*Co-expression correlation of FAM118A and HEXIM1*

Co-expression correlation analysis comparing *FAM118A* and *HEXIM1* in immune-related tissues pointed out to an involvement of these genes in methylation regulation processes. Epigenetic regulation is a complex mechanism of transcriptional modulation that can directly influence the immune response to viral or bacterial pathogens. One of the most common epigenetic modifications induced by bacterial components is histone acetylation/deacetylation. Equally, the interaction between viral pathogens and host cells can disrupt these epigenetic mechanisms, facilitating viral spread and an inadequate host immune response accompanied by inflammation, oxidative stress, apoptosis and organ dysfunction ^51;52^. It has been previously reported dysregulation of host epigenome in respiratory infections caused by certain viruses ^53-55^. Virus, such us highly virulent highly pathogenic avian influenza or MERS-CoV, modify histone methylation pattern to produce an under-regulation of the antiviral interferon induced genes impairing the host immune response against the infection ^53^. SARS-CoV-2 can also alter host epigenetic pathways affecting the expression of ACE2 and other genes with an important immuno-regulatory role ^56;57^. Concerning pneumonia condition, an involvement of chromatin remodeling pathways in the pathophysiology, severity and survival of CAP has been inferred from blood whole transcriptome data ^58^.

**5. Limitations**

There are several limitations in the present study. The most important one is that our pneumonia cohort is highly heterogeneous from the point of view of causal pathogen. Therefore, it is likely that our study lacks statistical power to detect genome susceptibility factors specific to particular viral or bacterial pneumonia. In turn, our findings, if confirmed, would represent susceptibility factors that are common to more general biological processes affecting pneumonia in general, independently of the causal agent involved. We are also uncertain about the connection between our top susceptibility genes with the age of patients, since we found suggestive links of association in adults and children with pneumonia. Also, if the present findings are corroborated in future studies, it would be also interesting to disentangle why some genes appear as statistically associated with severe phenotypes in some pneumonia patients (e.g. *HEXIM1* in severe COVID-19) but with milder forms of the disease in other (e.g. our cohort of patients with mild/moderate forms of pneumonia).

Genetic background could have some impact on gene expression, despite the fact that the vast majority of gene expression studies ignore this issue ^59^. However, there is a lack of information on the genetic background of the gene expression data used in the present study. While we know our COVID-19 cases have a main European genetic background as inferred from exome data (see ^49^ and unpublished data), there is no information for the reference controls. Additionally, inference is not possible for the microarray data obtained from GEO, which unfortunately precludes the possibility of inferring genetic background. This possibility would only be possible if RNA-seq data were available ^49^. The only information available regarding GEO datasets indicates that controls and cases were matched for “sex and race” in GSE103119, and “matched demographically and clinically” for GSE42834; with no info for GSE40012 and GSE65682. Note however that, the possible different genetic backgrounds in datasets are diluted/minimized when carrying out a meta-analysis because we used batch corrections in cases and controls. Therefore, although we cannot provide detailed information on genetic background of cohorts, there are solid guaranties that background noise is unlikely to alter the statistical findings in gene expression analysis.

**6. Final remarks**

Community-acquired pneumonia (CAP) is one of the main causes of hospitalization and mortality involving infectious diseases. With the aim of investigating the genetic susceptibility causes underlying CAP, we carried out a multi-centric and prospective study involving the recruitment of 300 adults with pneumonia and 438 healthy controls. Whole exome-wide sequencing data allowed to pinpoint a few SNP candidates (with rs1056322 being the most statistically significant in a recessive genetic model; *P*-value = 4.71×10^-6^, OR = 2.64) falling in a linkage disequilibrium region covering the *FAM118A* gene. A SKAT-O gene-based association test highlighted five additional gene candidates, namely *COL13A1*, *C20orf96*, *HEXIM1*, and *NPAS4*, and *STRC*. By meta-analyzing gene expression data from public repositories, we found several disease scenarios where these top genes are significantly dysregulated in non-COVID-19 pneumonia and in severe COVID-19 pneumonia patients. Remarkably, in COVID-19 patients, while the expression of *FAM118A* and *COL13A1* decay with the time from symptoms onset, the expression of *HEXIM1* shows the opposite trend. Predictive performances were assessed using area under the receiver operating characteristic curve (AUC-ROC), showing the predictive power of different combinations of the best candidate genes to predict the disease outcome. We found strong supportive genetic evidence underlying pneumonia outcome beyond its etiology.

**7. References**

1. Torres A, Peetermans WE, Viegi G, Blasi F. Risk factors for community-acquired pneumonia in adults in Europe: a literature review. *Thorax.* 2013;68(11):1057-1065.

2. Juvén T, Mertsola J, Waris M, et al. Etiology of community-acquired pneumonia in 254 hospitalized children. *Pediatr Infect Dis J.* 2000;19(4):293-298.

3. Butler-Laporte G, Povysil G, Kosmicki JA, et al. Exome-wide association study to identify rare variants influencing COVID-19 outcomes: Results from the Host Genetics Initiative. *PLoS Genet.* 2022;18(11):e1010367.

4. Salas A, Pardo-Seco J, Barral-Arca R, et al. Whole Exome Sequencing identifies new host genomic susceptibility factors in empyema caused by *Streptococcus pneumoniae* in children: A pilot study. *Genes (Basel).* 2018;9(5).

5. Martinon-Torres F, Png E, Khor CC, et al. Natural resistance to Meningococcal Disease related to CFH loci: Meta-analysis of genome-wide association studies. *Sci Rep.* 2016;6:35842.

6. Waterer GW, Wunderink RG. Genetic susceptibility to pneumonia. *Clin Chest Med.* 2005;26(1):29-38.

7. Chapman SJ, Vannberg FO, Khor CC, et al. Functional polymorphisms in the FCN2 gene are not associated with invasive pneumococcal disease. *Mol Immunol.* 2007;44(12):3267-3270.

8. Chapman SJ, Khor CC, Vannberg FO, et al. Common NFKBIL2 polymorphisms and susceptibility to pneumococcal disease: a genetic association study. *Crit Care.* 2010;14(6):R227.

9. Anderson D, Fakiola M, Hales BJ, Pennell CE, Thomas WR, Blackwell JM. Genome-wide association study of IgG1 responses to the choline-binding protein PspC of Streptococcus pneumoniae. *Genes Immun.* 2015;16(5):289-296.

10. Campos AI, Kho P, Vazquez-Prada KX, et al. Genetic Susceptibility to Pneumonia: A GWAS Meta-Analysis Between the UK Biobank and FinnGen. *Twin Res Hum Genet.* 2021;24(3):145-154.

11. Chen HH, Shaw DM, Petty LE, et al. Host genetic effects in pneumonia. *Am J Hum Genet.* 2021;108(1):194-201.

12. Gomez-Carballa A, Pardo-Seco J, Pischedda S, et al. Sex-biased expression of the TLR7 gene in severe COVID-19 patients: Insights from transcriptomics and epigenomics. *Environ Res.* 2022;215(Pt 2):114288.

13. Zhang Q, Bastard P, Effort CHG, Cobat A, Casanova JL. Human genetic and immunological determinants of critical COVID-19 pneumonia. *Nature.* 2022;603(7902):587-598.

14. Molina J, Gonzalez-Gamarra A, Ginel L, et al. CAPPRIC Study-Characterization of Community-Acquired Pneumonia in Spanish Adults Managed in Primary Care Settings. *Microorganisms.* 2021;9(3).

15. Cirulli ET, Lasseigne BN, Petrovski S, et al. Exome sequencing in amyotrophic lateral sclerosis identifies risk genes and pathways. *Science.* 2015;347(6229):1436-1441.

16. Genetics of Exfoliation Syndrome P, Li Z, Wang Z, et al. Association of rare CYP39A1 variants with exfoliation syndrome involving the anterior chamber of the eye. *JAMA.* 2021;325(8):753-764.

17. Salas A, Pardo-Seco J, Cebey-López M, et al. Whole Exome Sequencing reveals new candidate genes in host genomic susceptibility to Respiratory Syncytial Virus Disease. *Sci Rep.* 2017;7(1):15888.

18. Alexander DH, Novembre J, Lange K. Fast model-based estimation of ancestry in unrelated individuals. *Genome Res.* 2009;19(9):1655-1664.

19. Lee S, Emond MJ, Bamshad MJ, et al. Optimal unified approach for rare-variant association testing with application to small-sample case-control whole-exome sequencing studies. *Am J Hum Genet.* 2012;91(2):224-237.

20. Rentzsch P, Schubach M, Shendure J, Kircher M. CADD-Splice-improving genome-wide variant effect prediction using deep learning-derived splice scores. *Genome Med.* 2021;13(1):31.

21. Chang CC, Chow CC, Tellier LC, Vattikuti S, Purcell SM, Lee JJ. Second-generation PLINK: rising to the challenge of larger and richer datasets. *Gigascience.* 2015;4:7.

22.  *R: A Language and Enviroment for Statistical Computing* [computer program]. Vienna, Austria: R Foundation for Statistical Computing; 2019.

23. Pruim RJ, Welch RP, Sanna S, et al. LocusZoom: regional visualization of genome-wide association scan results. *Bioinformatics.* 2010;26(18):2336-2337.

24. *Genetic Data Handling (QC, GRM, LD, PCA) & Linear Mixed Models* [computer program]. R package version 1.5.7; 2020.

25. *SKAT: SNP-Set (Sequence) Kernel Association Test - R package version 2.2.5* [computer program]. 2023.

26. Scicluna BP, Klein Klouwenberg PM, van Vught LA, et al. A molecular biomarker to diagnose community-acquired pneumonia on intensive care unit admission. *Am J Respir Crit Care Med.* 2015;192(7):826-835.

27. Bloom CI, Graham CM, Berry MP, et al. Transcriptional blood signatures distinguish pulmonary tuberculosis, pulmonary sarcoidosis, pneumonias and lung cancers. *PLoS One.* 2013;8(8):e70630.

28. Parnell GP, McLean AS, Booth DR, et al. A distinct influenza infection signature in the blood transcriptome of patients with severe community-acquired pneumonia. *Crit Care.* 2012;16(4):R157.

29. Wallihan RG, Suarez NM, Cohen DM, et al. Molecular Distance to Health Transcriptional Score and Disease Severity in Children Hospitalized With Community-Acquired Pneumonia. *Front Cell Infect Microbiol.* 2018;8:382.

30. Carvalho BS, Irizarry RA. A framework for oligonucleotide microarray preprocessing. *Bioinformatics.* 2010;26(19):2363-2367.

31. Ritchie ME, Phipson B, Wu D, et al. *limma* powers differential expression analyses for RNA-sequencing and microarray studies. *Nucleic Acids Res.* 2015;43(7):e47.

32. Sweeney TE, Braviak L, Tato CM, Khatri P. Genome-wide expression for diagnosis of pulmonary tuberculosis: a multicohort analysis. *Lancet Respir Med.* 2016;4(3):213-224.

33. Jackson H, Rivero-Calle I, Broderick C, et al. Characterisation of the blood RNA host response underpinning severity in COVID‑19 patients. *Sci Rep.* 2022;12(1):12216.

34. Robin X, Turck N, Hainard A, et al. pROC: an open-source package for R and S+ to analyze and compare ROC curves. *BMC Bioinform.* 2011;12:77.

35. Miller HE, Bishop AJR. Correlation AnalyzeR: functional predictions from gene co-expression correlations. *BMC Bioinformatics.* 2021;22(1):206.

36. Slomian D, Szyda J, Dobosz P, et al. Better safe than sorry-Whole-genome sequencing indicates that missense variants are significant in susceptibility to COVID-19. *PLoS One.* 2023;18(1):e0279356.

37. Piñero J, Bravo A, Queralt-Rosinach N, et al. DisGeNET: a comprehensive platform integrating information on human disease-associated genes and variants. *Nucleic Acids Res.* 2017;45(D1):D833-D839.

38. Yuan Y, Zhou Y, Li Y, et al. Deconvolution of RNA-Seq Analysis of Hyperbaric Oxygen-Treated Mice Lungs Reveals Mesenchymal Cell Subtype Changes. *Int J Mol Sci.* 2020;21(4).

39. Saygili E, Devamoglu U, Goker-Bagca B, et al. A drug-responsive multicellular human spheroid model to recapitulate drug-induced pulmonary fibrosis. *Biomed Mater.* 2022;17(4).

40. Yap CX, Sidorenko J, Wu Y, et al. Dissection of genetic variation and evidence for pleiotropy in male pattern baldness. *Nat Commun.* 2018;9(1):5407.

41. Ellinghaus D, Jostins L, Spain SL, et al. Analysis of five chronic inflammatory diseases identifies 27 new associations and highlights disease-specific patterns at shared loci. *Nat Genet.* 2016;48(5):510-518.

42. Gardinassi LG. A Cross-Study Biomarker Signature of Human Bronchial Epithelial Cells Infected with Respiratory Syncytial Virus. *Adv Virol.* 2016;2016:3605302.

43. Morchikh M, Cribier A, Raffel R, et al. HEXIM1 and NEAT1 Long Non-coding RNA Form a Multi-subunit Complex that Regulates DNA-Mediated Innate Immune Response. *Mol Cell.* 2017;67(3):387-399 e385.

44. Kohoutek J, Blazek D, Peterlin BM. Hexim1 sequesters positive transcription elongation factor b from the class II transactivator on MHC class II promoters. *Proc Natl Acad Sci U S A.* 2006;103(46):17349-17354.

45. Price DH. P-TEFb, a cyclin-dependent kinase controlling elongation by RNA polymerase II. *Mol Cell Biol.* 2000;20(8):2629-2634.

46. Yik JH, Chen R, Pezda AC, Samford CS, Zhou Q. A human immunodeficiency virus type 1 Tat-like arginine-rich RNA-binding domain is essential for HEXIM1 to inhibit RNA polymerase II transcription through 7SK snRNA-mediated inactivation of P-TEFb. *Mol Cell Biol.* 2004;24(12):5094-5105.

47. Yoshikawa N, Shimizu N, Maruyama T, et al. Cardiomyocyte-specific overexpression of HEXIM1 prevents right ventricular hypertrophy in hypoxia-induced pulmonary hypertension in mice. *PLoS One.* 2012;7(12):e52522.

48. Zhu Z, Guo Y, Shi H, et al. Shared genetic and experimental links between obesity-related traits and asthma subtypes in UK Biobank. *J Allergy Clin Immunol.* 2020;145(2):537-549.

49. Gómez-Carballa A, Rivero-Calle I, Pardo-Seco J, et al. A multi-tissue study of immune gene expression profiling highlights the key role of the nasal epithelium in COVID-19 severity. *Environ Res.* 2022;210:112890.

50. Alpuche-Lazcano SP, Saliba J, Costa VV, et al. Profound downregulation of neural transcription factor Npas4 and Nr4a family in fetal mice neurons infected with Zika virus. *PLoS Negl Trop Dis.* 2021;15(5):e0009425.

51. Crimi E, Benincasa G, Figueroa-Marrero N, Galdiero M, Napoli C. Epigenetic susceptibility to severe respiratory viral infections and its therapeutic implications: a narrative review. *Br J Anaesth.* 2020;125(6):1002-1017.

52. Bierne H, Hamon M, Cossart P. Epigenetics and bacterial infections. *Cold Spring Harb Perspect Med.* 2012;2(12):a010272.

53. Menachery VD, Eisfeld AJ, Schafer A, et al. Pathogenic influenza viruses and coronaviruses utilize similar and contrasting approaches to control interferon-stimulated gene responses. *mBio.* 2014;5(3):e01174-01114.

54. Yang X, Rutkovsky AC, Zhou J, et al. Characterization of Altered Gene Expression and Histone Methylation in Peripheral Blood Mononuclear Cells Regulating Inflammation in COVID-19 Patients. *J Immunol.* 2022;208(8):1968-1977.

55. Wang K, Deng H, Song B, et al. The Correlation Between Immune Invasion and SARS-COV-2 Entry Protein ADAM17 in Cancer Patients by Bioinformatic Analysis. *Front Immunol.* 2022;13:923516.

56. Morselli M, Farrell C, Montoya D, et al. DNA methylation profiles in pneumonia patients reflect changes in cell types and pneumonia severity. *Epigenetics.* 2022;17(12):1646-1660.

57. Li S, Ma F, Yokota T, et al. Metabolic reprogramming and epigenetic changes of vital organs in SARS-CoV-2-induced systemic toxicity. *JCI Insight.* 2021;6(2).

58. Hopp L, Loeffler-Wirth H, Nersisyan L, Arakelyan A, Binder H. Footprints of Sepsis Framed Within Community Acquired Pneumonia in the Blood Transcriptome. *Front Immunol.* 2018;9:1620.

59. Barral-Arca R, Pardo-Seco J, Bello X, Martinón-Torres F, Salas A. Ancestry patterns inferred from massive RNAseq data. *RNA.* 2019;25(7):857-868.

**Figure S1. Allele association test.** Manhattan plot (A) and QQ-plot (B) of computed *P*-values for the single allelic test.


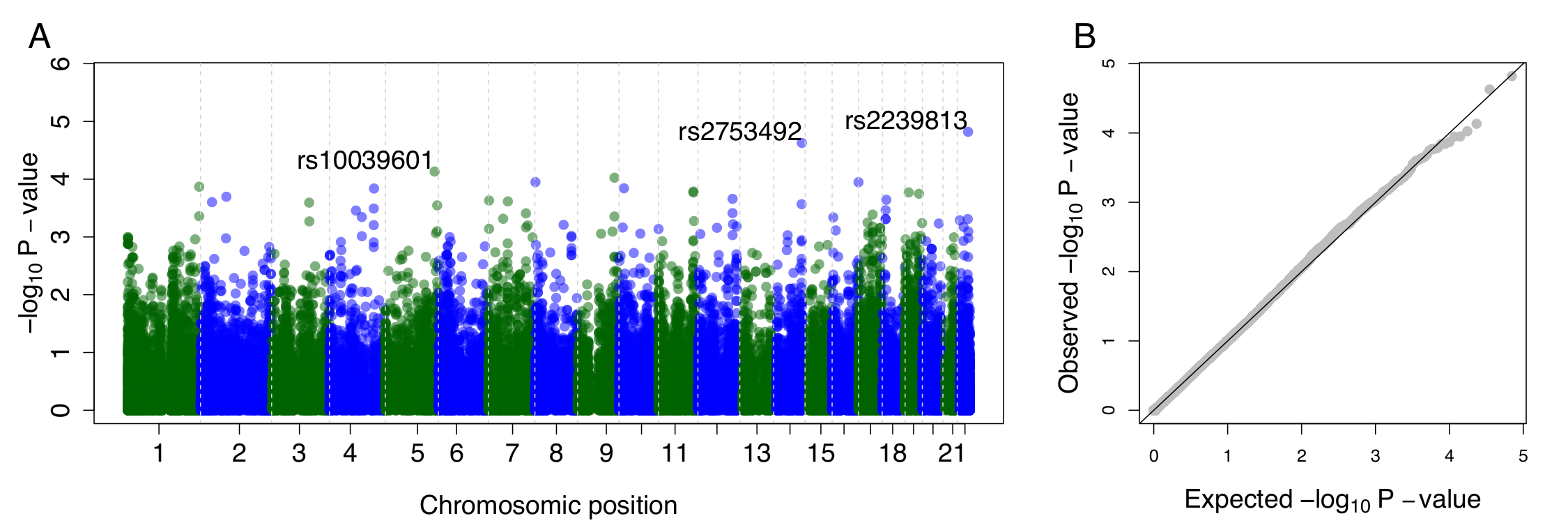


**Figure S2. Association tests for genotype test models.** Manhattan plot (A, C, E) and QQ-plots (B, D, F) of computed *P*-values for the genotyping test models (additive, dominant, and recessive models; respectively).


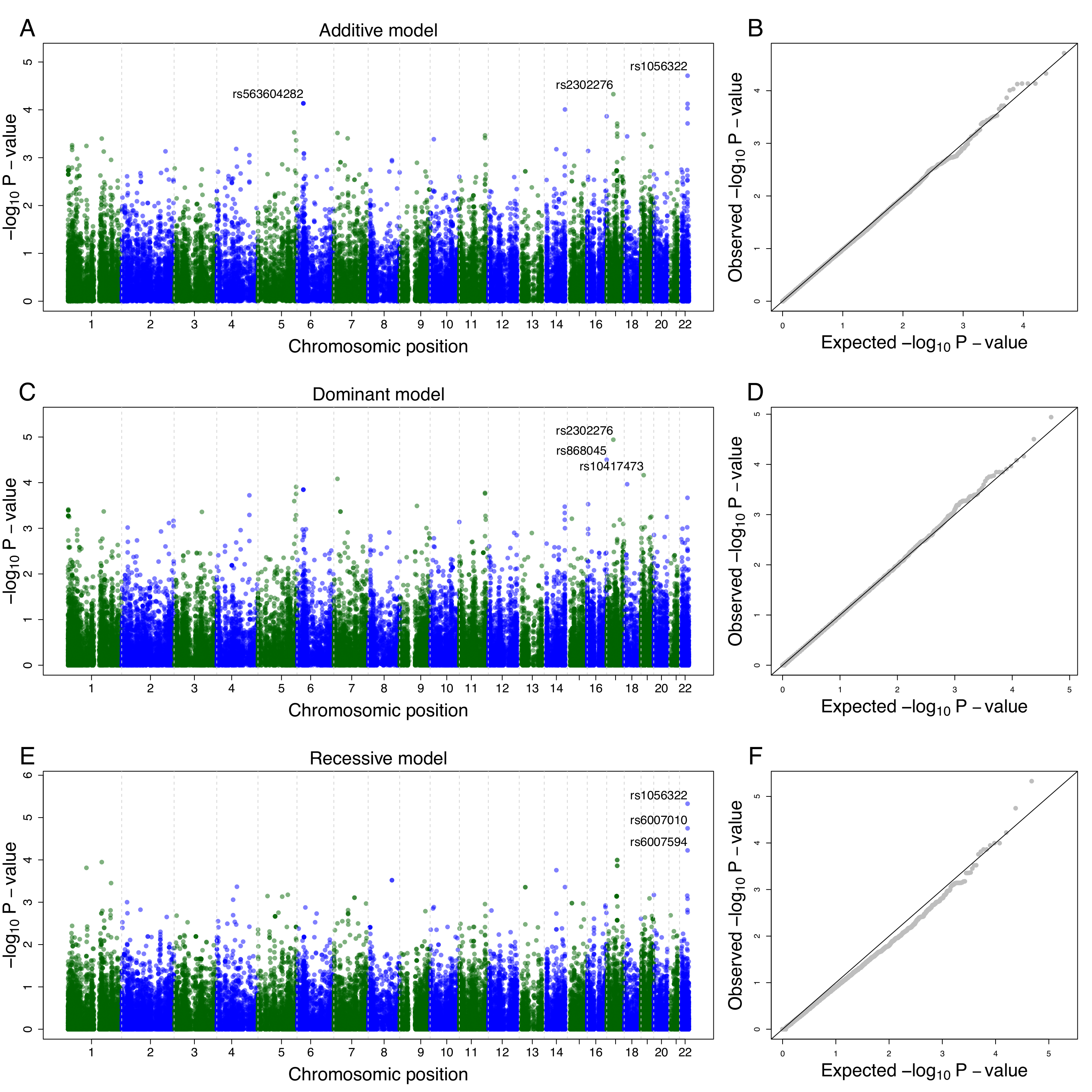


**Figure S3. *P*-value and LD plot (measured as *r*^2^) for rs1056322, the best candidate SNP in the recessive genotype model.** This SNP is used as reference for the computation of LD values in the plot.


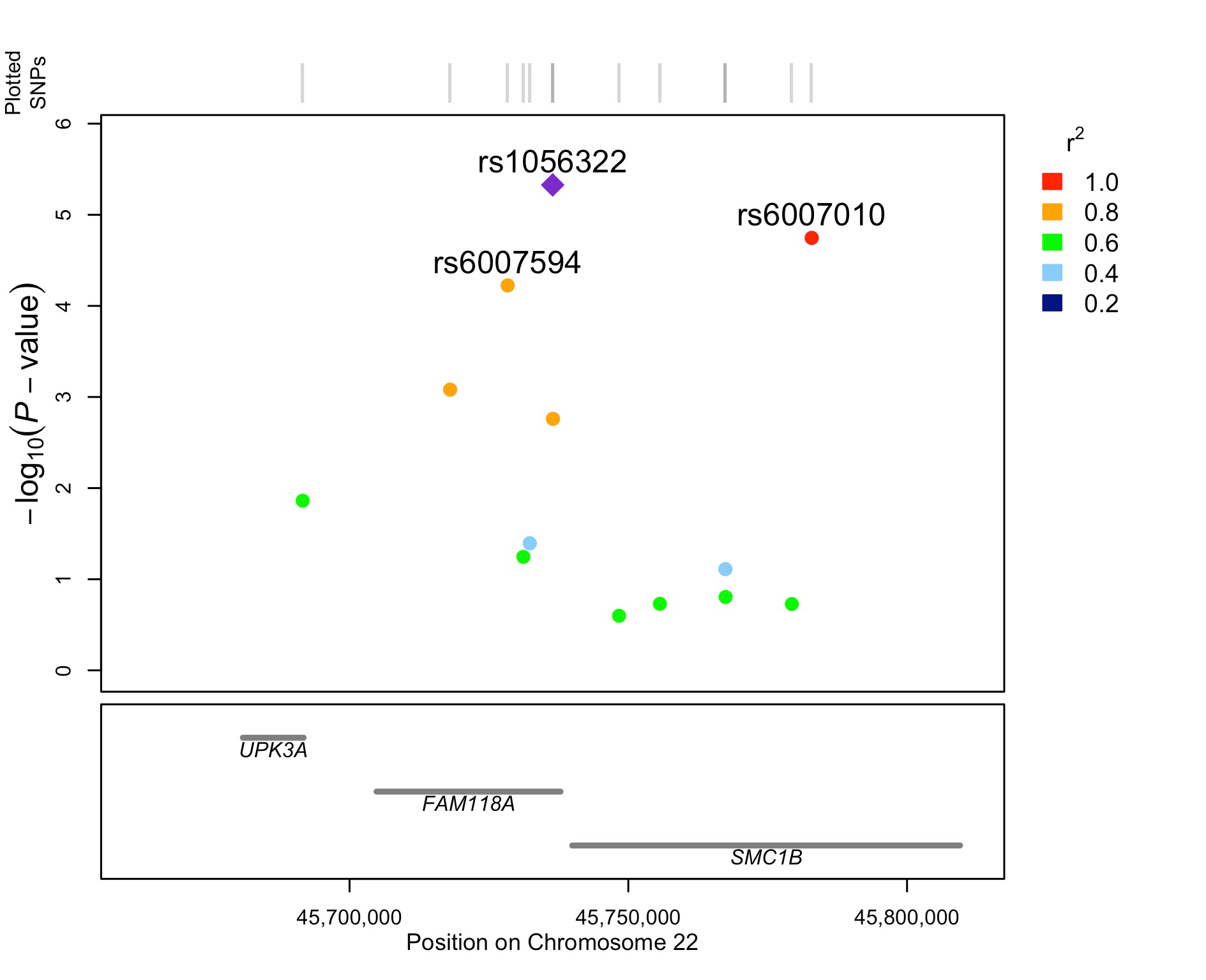


**Figure S4. Pairwise LD plot (measured as *r*^2^) for the region around the SNP rs1056322.**


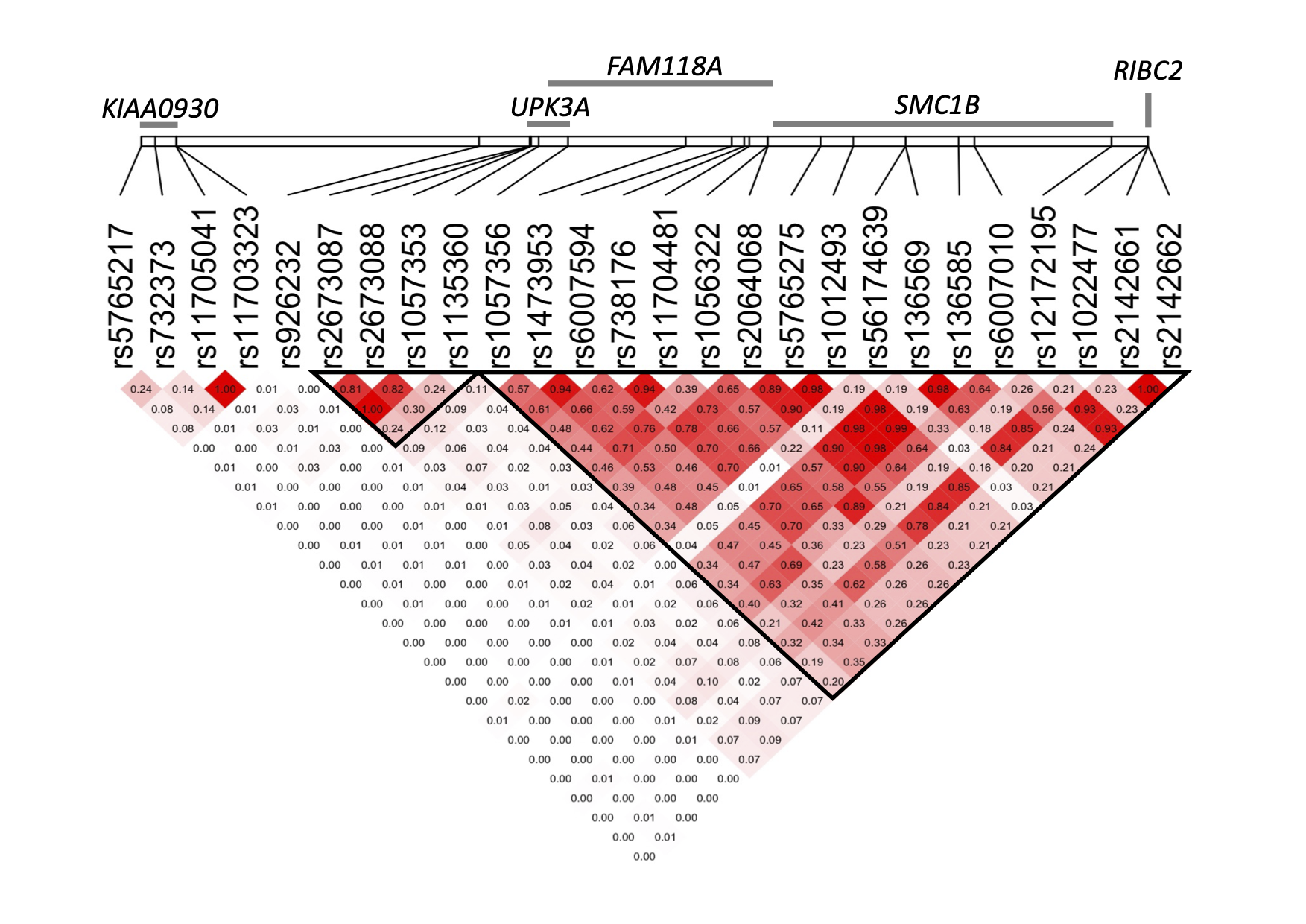


**Figure S5**. **Heatmap of top differentially and top similar** **correlated pathways detected from FAM118A and HEXIM1 in immune-related tissues and using biological processes terms from GO (Gene ontology) as reference database.** NES: normalized enrichment score.
