# Supplementary material for: Whole exome sequencing identifies new susceptibility candidates underlying community-acquired pneumonia
Source: Genes Dis. 2023 Nov 19;11(6):101170. doi: 10.1016/j.gendis.2023.101170 (PMC11327392; doi:10.1016/j.gendis.2023.101170)
Supplement: Multimedia component 2 [file mmc2.docx]

**Table S1**. Demographic and clinical characteristics of the CAP study cohort. Variables are displayed as percentages (number of patients in round brackets), except for the age variable that is shown as median (IQR).

| **Variable** | **CAP**  **(*n* = 300)** |
| --- | --- |
| Age | 58 (39.2-70.0) |
| Sex (female) | 49.3% (148) |
| **Medical history** |  |
| COPD | 8.7% (26) |
| Asthma | 7.3% (22) |
| Cardiac insufficiency | 3.3% (10) |
| Coronary disease | 4.0% (12) |
| Brain disease | 2.3% (7) |
| Diabetes | 4% (12) |
| Pneumococcal vaccine | 18.9% (55) |
| Flu vaccine | 37.7% (110) |
| **Diagnosis** |  |
| Fever | 72.3% (217) |
| Hypothermia | 0.7% (2) |
| Chills | 38.0% (144) |
| Thorax pain | 36.3% (109) |
| Cough | 87.7% (263) |
| Expectoration | 52.7% (158) |
| Dyspnea | 31.7% (95) |
| Tachypnea | 2.0% (6) |
| Upset | 64.3% (193) |
| Crackles or consoled pulmonary | 56.0% (168) |
| **Clinical course** |  |
| Hospitalization | 2.0% (6) |
| Severe outcome | 2.0% (6) |
| **Radiologic findings** |  |
| Pleural effusion | 4.7% (14) |
| Pulmonary density increasing | 77.3% (232) |
| Alveolar infiltrates | 31.7% (95) |
| Multi-lobar | 2.0% (6) |
| Lobular | 19.3% (58) |
| Segmental | 10.3% (31) |
