# Supplementary material for: Whole exome sequencing identifies new susceptibility candidates underlying community-acquired pneumonia
Source: Genes Dis. 2023 Nov 19;11(6):101170. doi: 10.1016/j.gendis.2023.101170 (PMC11327392; doi:10.1016/j.gendis.2023.101170)
Supplement: Multimedia component 3 [file mmc3.docx]

**Table S2.** Gene association test for the SKAT-O model considering whole gene variability and only the rare variants (MAF < 0.05). Only genes having at least 10 SNPs were considered. For each model, we displayed the genes surpassing the FDR correction threshold.

| **Gene** | **Nº of markers** | ***P*-value** | **FDR *P*-value** | |
| --- | --- | --- | --- | --- |
| SKAT-O all variants | | | |  |
| *C20orf96* | 35 | 9.55×10^-6^ | 4.89×10^-2^ |  |
| *COL13A1* | 102 | 2.43×10^-6^ | 2.61×10^-2^ |  |
| *HEXIM1* | 21 | 6.18×10^-6^ | 4.42×10^-2^ |  |
| *NPAS4* | 24 | 1.14×10^-5^ | 4.89×10^-2^ |  |
| *STRC* | 58 | 6.95×10^-7^ | 1.49×10^-2^ |  |
| SKAT-O rare variants | | | |  |
| *C20orf96* | 25 | 1.15×10^-5^ | 4.90×10^-2^ |  |
| *COL13A1* | 85 | 8.03×10^-7^ | 1.35×10^-2^ |  |
| *HEXIM1* | 17 | 5.17×10^-6^ | 3.43×10^-2^ |  |
| *NPAS4* | 22 | 1.23×10^-5^ | 4.90×10^-2^ |  |
| *STRC* | 47 | 1.35×10^-6^ | 1.35×10^-2^ |  |
