# Supplementary material for: Whole exome sequencing identifies new susceptibility candidates underlying community-acquired pneumonia
Source: Genes Dis. 2023 Nov 19;11(6):101170. doi: 10.1016/j.gendis.2023.101170 (PMC11327392; doi:10.1016/j.gendis.2023.101170)
Supplement: Multimedia component 4 [file mmc4.docx]

**Table S3.** Statistical significance for the differential expression between cases and healthy controls for the best gene candidates. GEO or ArrayExpress (AE) accession numbers and sample sizes are indicated (in round brackets: number of cases | number of controls). In the COVID-19 dataset we only used the severity category (WHO score > 5).

|  | *FAM118A* | *COL13A1* | *STRC* | *C20orf96* | *NPAS4* | *HEXIM1* |
| --- | --- | --- | --- | --- | --- | --- |
| ***Non COVID-19 adults*** |  |  |  |  |  |  |
| GEO: GSE65682 (194\|40) | **1.7×10^-13^** | **2.3×10^-02^** | 1.0 | **9.9×10^-02^** | **2.5×10^-04^** | 0.23 |
| GEO: GSE42834 (6\|113) | **6.2×10^-02^** | 0.98 | 0.31 | 0.34 | 0.32 | 0.77 |
| GEO: GSE40012 (224\|18) | 0.52 | 0.73 | **2.6×10^-02^** | 0.34 | **4.1×10^-02^** | 0.91 |
| Meta-analysis (437\|171) | **2.9×10^-05^** | 0.74 | 0.42 | **2.0×10^-04^** | **8×10^-06^** | 0.86 |
| ***Non COVID-19 pediatric*** |  |  |  |  |  |  |
| GEO: GSE103119 (152\|20) | **2.3×10^-03^** | 0.26 | **1.0×10^-03^** | 0.11 | 0.87 | 0.32 |
| ***Severe COVID-19 adults*** |  |  |  |  |  |  |
| AE: MTAB-10926 (7\|10) | **5.7×10^-02^** | **4.5×10^-02^** | – | 0.26 | – | **2.2×10^-02^** |
